# Comparison of Colorimetric (Enzyme-Linked Immunosorbent Assay (ELISA)) and Chemiluminescent Immunoassay (CLIA) Methods for Detection of Dengue Nonstructural Protein 1 (NS1) Antigen in Acute Dengue Infection

**DOI:** 10.7759/cureus.109055

**Published:** 2026-05-17

**Authors:** Fatima Masood, Afsheen Nigar, Rukhsana Tumrani, Madiha Naqvi, Zainab Awais, Hassan R Heral, Ayesha Masood, Rehma Dar, Maham Shakoor, Farheen Ansari

**Affiliations:** 1 Pathology, University College of Medicine and Dentistry, University of Lahore, Lahore, PAK; 2 Pathology, King Edward Medical University, Lahore, PAK; 3 Pathology, Shalamar Medical and Dental College, Lahore, PAK; 4 Pathology, Faryal Dental College, Lahore, PAK; 5 Pathology, Gujaranwala Medical College, Lahore, PAK; 6 Pathology, Akhtar Saeed Medical and Dental College, Lahore, PAK; 7 Microbiology, Institute of Molecular Biology and Biotechnology, University of Lahore, Lahore, PAK

**Keywords:** clia, dengue, diagnostic accuracy, elisa, ns1 antigen, real-time pcr

## Abstract

Background: Early diagnosis of dengue virus infection is essential for timely clinical management. Detection of nonstructural protein 1 (NS1) antigen plays an important role during the acute phase of illness. This study aimed to compare the diagnostic performance of enzyme-linked immunosorbent assay (ELISA) and chemiluminescence immunoassay (CLIA) for NS1 antigen detection in acute dengue infection.

Methods: This cross-sectional comparative study included 50 patients with suspected acute dengue infection. Serum samples were collected at admission (day 0) and on days 2 and 4 of illness. Dengue infection was confirmed using real-time polymerase chain reaction (RT-PCR) as the reference standard. NS1 antigen detection was performed using NOVATEINBIO ELISA and MAGLUMI® CLIA assays. Dengue IgM and IgG antibodies were also assessed using ELISA. Sensitivity, specificity, positive predictive value, negative predictive value, and diagnostic accuracy were calculated for both methods.

Results: RT-PCR confirmed dengue infection in 39 (78%) of cases. NS1 ELISA demonstrated a sensitivity of 89.74% and specificity of 81.81%, whereas CLIA showed higher sensitivity (94.87%) and specificity (90.90%). NS1 antigen levels were highest during early illness and declined by day 4. IgM antibodies became detectable in 33 patients by day 4, while IgG antibodies remained negative. Hematological findings demonstrated progressive leukopenia and thrombocytopenia.

Conclusion: CLIA demonstrated higher sensitivity and specificity compared with ELISA for NS1 antigen detection. Its automation and improved performance make it a valuable tool for the early diagnosis of dengue infection in endemic settings.

## Introduction

Dengue fever is one of the most common mosquito-borne viral infections worldwide and is highly prevalent in tropical and subtropical regions [1]. Pakistan has experienced recurrent dengue epidemics, posing a substantial burden on the healthcare system [2]. Early laboratory diagnosis of dengue virus infection is essential for appropriate clinical management, timely intervention, and reduction of morbidity and mortality [3].

Dengue virus nonstructural protein 1 (NS1) is secreted into the bloodstream during the early phase of infection, making it a valuable biomarker for early diagnosis [4]. The NS1 antigen is detectable during the early febrile phase and is particularly useful before the appearance of dengue-specific antibodies [5]. Enzyme-linked immunosorbent assay (ELISA) has traditionally been used for NS1 antigen detection; however, newer chemiluminescence-based immunoassays have been introduced with potential advantages in sensitivity, specificity, and automation [6].

Despite the increasing availability of chemiluminescence immunoassays (CLIA), limited local data are available comparing their diagnostic performance with conventional ELISA for NS1 antigen detection. Accurate and early detection of dengue infection is particularly important in endemic regions where rapid diagnosis may improve patient management and reduce disease-related complications.

Therefore, this study was conducted to compare the diagnostic performance of ELISA and CLIA for the detection of dengue NS1 antigen using real-time polymerase chain reaction (RT-PCR) as the reference standard.

## Materials and methods

Study design and setting

This cross-sectional comparative study was conducted at the Institute of Molecular Biology and Biotechnology, in collaboration with the Department of Chemical Pathology, at a tertiary care teaching hospital in Pakistan.

Sample size calculation

The sample size was calculated using the standard formula for diagnostic studies: n=Z²×P×(1-P)/d², where Z=1.96 at a 95% confidence level, P is the expected sensitivity, and d is the absolute precision. Based on previous studies reporting NS1 antigen sensitivity ranging between 70% and 90% in early dengue infection, an expected sensitivity of 85% was assumed [7]. With a precision of 10%, the calculated sample size was 49, which was rounded to 50.

Study duration

The study was carried out over a period of six months from October 1, 2023, to March 31, 2024.

Study population

Adult patients of either gender presenting with clinical suspicion of acute dengue infection were enrolled after obtaining written informed consent.

Inclusion criteria

Patients aged 18-50 years presenting during the early febrile phase of suspected acute dengue infection, with or without clinical features of dengue-associated hemorrhagic manifestations, including petechiae, mucosal bleeding, or thrombocytopenia, were included in the study.

Exclusion criteria

Patients with confirmed alternative febrile illnesses, chronic hematological disorders, known coagulation abnormalities, or inadequate blood samples were excluded from the study.

Sample collection and laboratory procedures

Blood samples were collected under aseptic conditions at admission (day 0) and on days 2 and 4 of illness. Serum was separated and processed for laboratory analysis.

Dengue virus infection was confirmed using RT-PCR with the HELINI Dengue Real-Time PCR kit (HELINI Biomolecules, India), which served as the reference standard.

NS1 antigen detection was performed on all samples using NOVATEINBIO Dengue NS1 ELISA kits and the MAGLUMI® CLIA assay system (Snibe Diagnostics, China). The diagnostic performance of ELISA and CLIA was compared against RT-PCR.

Dengue IgM and IgG antibodies were additionally assessed using ELISA in follow-up samples collected during the course of illness.

Statistical analysis

Data were analyzed using SPSS software version 27 (IBM Corp., Armonk, NY, USA). Sensitivity, specificity, positive predictive value (PPV), negative predictive value (NPV), and diagnostic accuracy were calculated for both NS1 detection methods using RT-PCR as the gold standard, according to the following formulae: sensitivity=TP/(TP+FN)×100; specificity=TN/(TN+FP)×100; positive predictive value (PPV)=TP/(TP+FP)×100; negative predictive value (NPV)=TN/(TN+FN)×100; and diagnostic accuracy=(TP+TN)/(TP+FP+FN+TN)×100.

Ethical approval

Ethical approval was obtained from the Institutional Review Board of Molecular Biology and Biotechnology (Ref. IMBB/BBBC/23/483-A), dated May 15, 2023, prior to initiation of the study.

## Results

The study included patients with suspected acute dengue infection who fulfilled the inclusion criteria. Dengue virus infection was confirmed by RT-PCR. Demographic characteristics of the study participants are summarized in Table 1.

**Table 1:** Demographic characteristics of study participants (n=50)

Variable	Value
Total patients	50
Males	27 (54%)
Females	23 (46%)
Age in years (mean±SD)	32.4±8.6
Duration of fever in days (mean±SD)	2.8±1.1

RT-PCR detected dengue viral RNA in 39 (78%) of the suspected cases, confirming acute dengue virus infection, while 11 (22%) samples were negative. The distribution of RT-PCR and NS1 antigen detection results is summarized in Table 2.

**Table 2:** Comparison of RT-PCR and NS1 antigen detection methods NS1, nonstructural protein 1; ELISA, enzyme-linked immunosorbent assay; CLIA, chemiluminescence immunoassay; RT-PCR, real-time polymerase chain reaction

Diagnostic test	Positive (n)	Negative (n)
RT-PCR	39	11
NS1 ELISA	37	13
NS1 CLIA	38	12

NS1 antigen detection by colorimetric ELISA and CLIA

NS1 antigen testing by both ELISA and CLIA was performed on all study samples (n=50) and compared with RT-PCR results as the reference standard. The diagnostic performance of both methods is summarized in Table 3.

**Table 3:** Diagnostic performance of ELISA and CLIA for NS1 antigen detection using RT-PCR as the reference standard (n=50) TP, true positive; FP, false positive; FN, false negative; TN, true negative; PPV, positive predictive value; NPV, negative predictive value; ELISA, enzyme-linked immunosorbent assay; CLIA, chemiluminescence immunoassay; RT-PCR, real-time polymerase chain reaction; NS1, nonstructural protein 1

Test	TP	FP	FN	TN	Sensitivity (%)	Specificity (%)	PPV (%)	NPV (%)	Accuracy (%)
ELISA	35	2	4	9	89.74	81.81	94.59	69.23	88
CLIA	37	1	2	10	94.87	90.9	97.36	83.33	94

Serological findings

IgM and IgG ELISA results demonstrated negative IgM and IgG findings on day 2 in most patients. On day 4 of illness, 33 patients were IgM-positive, with a mean absorbance of 2.261±0.015, while IgG antibodies remained negative in all tested cases. Quantitative NS1 values obtained by ELISA and CLIA are presented in Table 4.

**Table 4:** Quantitative NS1 values measured by ELISA and CLIA ELISA, enzyme-linked immunosorbent assay; CLIA, chemiluminescence immunoassay; NS1, nonstructural protein 1

Assay	Positive samples (mean±SD)	Negative samples (mean±SD)
NS1 ELISA (day 2)	4.78±0.69	0.058±0.007
NS1 ELISA (day 4)	2.06±0.005	-
NS1 CLIA (AU/mL)	8.6±0.64	0.27±0.052

The distribution of IgM and IgG serological results is summarized in Table 5.

**Table 5:** Serological findings (IgM and IgG ELISA) in study participants (n=50) ELISA, enzyme-linked immunosorbent assay

Parameter	Day 2 (n)	Day 4 (n)
IgM positive	0	33
IgM negative	50	17
IgG positive	0	0
IgG negative	50	50

Hematological findings

Hematological analysis showed a mean total leukocyte count (TLC) of 4.7±1.2 ×10⁹/L and a platelet count of 165±28 ×10⁹/L at presentation (day 0). On day 2 of illness, the mean TLC decreased to 3.5±0.72 ×10⁹/L, and the mean platelet count declined to 122±16 ×10⁹/L, reflecting disease progression.

Changes in hematological parameters during the course of illness are shown in Table 6.

**Table 6:** Hematological parameters at different time points

Parameter	Day 0 (mean±SD)	Day 2 (mean±SD)
Total leukocyte count (×10⁹/L)	4.7±1.2	3.5±0.72
Platelet count (×10⁹/L)	165±28	122±16

## Discussion

Early and accurate diagnosis of dengue infection is essential for timely clinical management and prevention of disease-related complications, particularly in endemic regions [3]. Detection of NS1 antigen during the acute febrile phase provides an important opportunity for early diagnosis before the development of detectable antibodies [4]. In the present study, the diagnostic performance of ELISA and CLIA for NS1 antigen detection was compared using RT-PCR as the reference standard.

The findings of this study demonstrated that CLIA exhibited higher sensitivity and specificity compared with ELISA for NS1 antigen detection. The sensitivity and specificity of CLIA were 94.87% and 90.90%, respectively, whereas ELISA demonstrated sensitivity and specificity of 89.74% and 81.81%, respectively. These findings suggest that CLIA may provide improved diagnostic performance during the early phase of dengue infection. Similar findings have been reported in previous studies demonstrating enhanced analytical sensitivity and automation associated with chemiluminescence-based assays [8].

The present study also demonstrated that NS1 antigen levels were highest during the initial days of illness and gradually declined by day 4. This observation is consistent with the known kinetics of dengue NS1 antigenemia and supports the utility of NS1-based assays for early diagnosis [9]. IgM antibodies became detectable in a significant number of patients by day 4, whereas IgG antibodies remained negative, indicating the predominance of acute primary infection among the studied participants.

Hematological findings in the present study demonstrated progressive leukopenia and thrombocytopenia during the course of illness. These findings are consistent with the hematological abnormalities commonly observed in acute dengue infection and may provide supportive clinical evidence during disease progression [10].

The automated nature of CLIA offers additional practical advantages, including reduced manual handling, improved workflow efficiency, and potentially shorter turnaround time compared with conventional ELISA methods [11,12]. These characteristics may be particularly beneficial in high-volume laboratories and dengue-endemic settings where rapid diagnosis is essential for patient management and outbreak control.

However, the interpretation of the findings should be considered in light of certain methodological limitations. The study was conducted at a single center with a relatively small sample size, which may limit the generalizability of the results. In addition, advanced statistical comparisons between the diagnostic methods were limited. Further multicenter studies with larger sample sizes are recommended to validate these findings and evaluate additional practical aspects such as cost-effectiveness, turnaround time, and implementation feasibility in routine clinical laboratories.

Overall, the findings of the present study support the potential utility of CLIA as a reliable and sensitive diagnostic tool for the early detection of dengue NS1 antigen compared with conventional ELISA.

## Conclusions

CLIA demonstrated higher sensitivity and specificity compared with ELISA for the detection of dengue NS1 antigen in acute dengue infection. The improved diagnostic performance and automated processing capabilities of CLIA suggest that it may serve as a valuable tool for early dengue diagnosis, particularly in endemic settings and high-throughput laboratories. Early NS1 antigen detection remains clinically important for the timely diagnosis and management of dengue infection. Further multicenter studies with larger sample sizes are recommended to confirm these findings and evaluate additional practical benefits, including turnaround time and cost-effectiveness.
